# Physiological and Proteomic Responses of Mulberry Trees (*Morus alba.* L.) to Combined Salt and Drought Stress

**DOI:** 10.3390/ijms20102486

**Published:** 2019-05-20

**Authors:** Yan Liu, Dongfeng Ji, Robert Turgeon, Jine Chen, Tianbao Lin, Jing Huang, Jie Luo, Yan Zhu, Cankui Zhang, Zhiqiang Lv

**Affiliations:** 1 Sericultural Research Institute, Zhejiang Academy of Agricultural Sciences, Hangzhou 310021, China; mayanly@sina.com (Y.L.); jdf6060@sohu.com (D.J.); chenje79@126.com (J.C.); kissumbra@126.com (T.L.); zhuy@mail.zaas.ac.cn (Y.Z.); 2 Plant Biology Section, School of Integrative Plant Science, Cornell University, Ithaca, NY 14853, USA; ert2@cornell.edu; 3 Department of Agronomy and Purdue Center for Plant Biology, Purdue University, West Lafayette, IN 47907, USA; huang877@purdue.edu; 4 Technology Support Center, Zhejiang Academy of Agricultural Science, Hangzhou 310021, China; luojie@mail.zaas.ac.cn

**Keywords:** mulberry, TMT proteomics, stressed roots, stressed leaves, sugar metabolism

## Abstract

Intensive investigations have been conducted on the effect of sole drought or salinity stress on the growth of plants. However, there is relatively little knowledge on how plants, particularly woody species, respond to a combination of these two stresses although these stresses can simultaneously occur in the field. In this study, mulberry, an economically important resource for traditional medicine, and the sole food of domesticated silkworms was subjected to a combination of salt and drought stress and analyzed by physiological methods and TMT-based proteomics. Stressed mulberry exhibited significant alteration in physiological parameters, including root/shoot ratio, chlorophyll fluorescence, total carbon, and ion reallocation. A total of 577 and 270 differentially expressed proteins (DEPs) were identified from the stressed leaves and roots, respectively. Through KEGG analysis, these DEPs were assigned to multiple pathways, including carbon metabolism, photosynthesis, redox, secondary metabolism, and hormone metabolism. Among these pathways, the sucrose related metabolic pathway was distinctly enriched in both stressed leaves and roots, indicating an important contribution in mulberry under stress condition. The results provide a comprehensive understanding of the adaptive mechanism of mulberry in response to salt and drought stress, which will facilitate further studies on innovations in terms of crop performance.

## 1. Introduction

Soil salinity and water deficit are the two most common abiotic stresses that constrain plant growth and productivity [1]. In the cytoplasm, salt inhibits many enzymatic processes, including photosynthesis, due to the imbalance of cellular K^+^/Na^+^ [2]. Dehydration reduces cell water availability, disrupts normal cellular activities, and compromises photosynthesis [3]. In past decades, intensive investigations of regulatory mechanisms under salt or drought stress have been conducted on many plants, including *Arabidopsis* [4], rice [5], wheat [6], *Malus* [7], maize [8] and cotton [9]. However, relatively little has been reported on intrinsic responses to a combination of two or more stressors, although this commonly occurs in the field. Plants confronted with a combination of stressors often produce unpredictable changes, and the involved signaling pathways cannot be directly extrapolated from the study of these stresses individually [10,11,12,13].

In plants, leaves (source tissues) and roots (sink tissues) are two distinct resource pools that are connected via the vascular system [14]. Photosynthetic processes in leaves are strongly dependent on water, mineral nutrients, hormones and proteins that are synthesized in or taken up by the roots, and conversely, resource uptake in roots is assured by photosynthetic assimilates transferred from shoots to roots [15]. When plants are grown under adverse environmental conditions, signals rapidly respond and function in different organs to help the plant to adapt [16,17]. Various signaling molecules, such as calcium, phytohormones, and mobile peptides, are important players in shoot to root, or vice versa, communication in plants under environmental stress [17]. However, systematic comparisons among leaves and roots and the possible metabolism between these organs under different growth environments are still limited. Understanding the integration of changes and possible inter-communication mechanisms is essential to increasing our knowledge of plant growth and development.

The mulberry (*Morus alba* L., Moraceae) tree is an economically and ecologically important perennial woody plant [18]. It is known for rapid growth and biomass production, active plant—microbe interactions, nutritional and medicinal value, and endogenous abiotic stress tolerance (including cold, waterlogging, drought, salinity, and heavy metal ions) [19]. These characteristics have made mulberry a promising perennial model system [20]. Recently, with the release of the *Morus notablilis* genome sequence, genome-wide transcriptomic and proteomic analyses can be applied to mulberry [21,22]. Transcriptome studies have been conducted in mulberry fruit in response to *Ciboria carunculoides* [23] and drought tolerance [24,25]. However, a comprehensive understanding of the mechanisms that mulberry trees regulate in different environmental conditions is lacking.

In the present study, we conducted an integrated physiological and TMT (tandem mass tag)-based proteomics analysis on mulberry seedlings in response to a combination of salt and drought stress for two weeks. Our objective was to gain a global view of the molecular mechanisms in mulberry to these stresses and identify key regulatory networks and proteins contributing to mulberry stress tolerance. Our discovery will lay the foundation for future genetic improvements to enhance mulberry productivity and adaptability to stress.

## 2. Results and Discussion

### 2.1. Physiological Responses of Mulberry to Salt and Drought Stress

**Figure 1:**
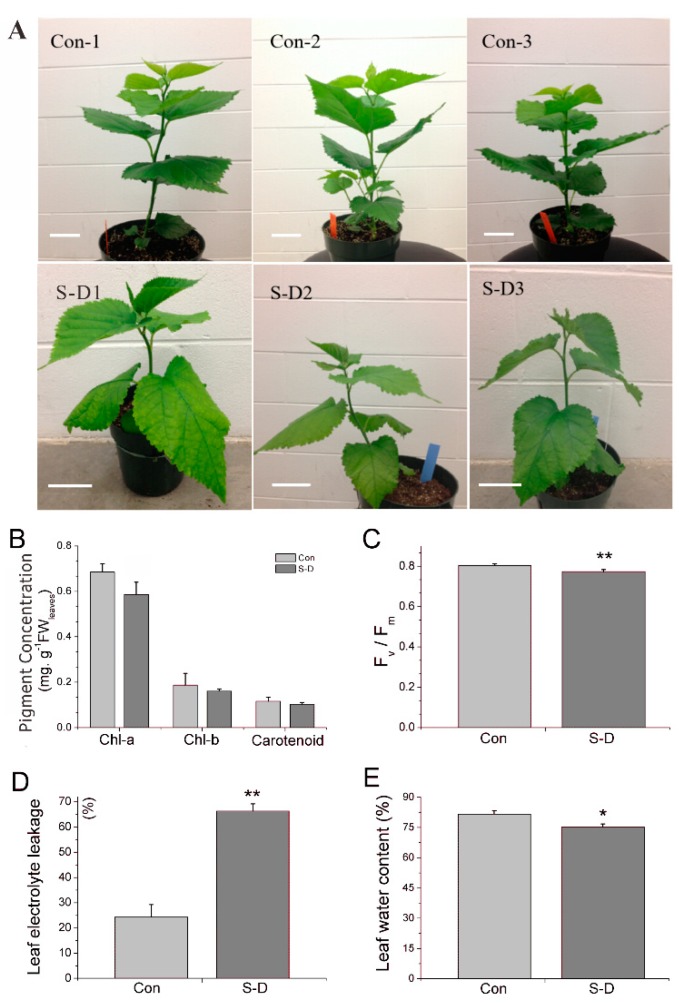
Physiological responses of mulberry to a combination of salt and drought stress. (**A**) Growth phenotype of mulberry seedlings at the end of a combination of salt and drought stress. These plants were treated with 200 mM NaCl for two days and continued dehydration for two weeks. Scale bar = 5 cm. (**B**) Chlorophyll content (Chl-a, Chl-b, and carotenoid, mg. g−1.FW) in both the control (Con) and salt-drought stress groups (S-D). (**C**) Chlorophyll fluorescence parameter F_v_/F_m_ in both Con and S-D group. (**D**) Leaf electrolyte leakage (%) in Con and S-D group. (**E**) Leaf water content (%) in both Con and S-D group. The values of each column are the means ± S.D. of three biological replicates. Asterisks labeled above the columns indicate a significant difference at the *p* ≤ 0.05 level by Duncan’s test using SPSS software (version 22.0).

After irrigation with 200 mM NaCl for two days and subsequent continuous dehydration for two weeks, the relative moisture content of soil decreased while its salinity increased distinctly in the salt-drought stress group (Figure S1). At the end of the treatment, in comparison to mulberry trees grown under normal conditions, trees grown under salt and drought stress conditions showed symptoms, such as dwarf and petiole droop (Figure 1). The root/shoot (R/S) biomass ratio increased from 0.12 under normal conditions to 0.26 under stressed conditions in mulberry (Table 1), which implied that plant resource (e.g., C and N) partitioning may have been prioritized to roots over shoots under salt-drought stress conditions, as occurred in previous reports on mastic trees [26] and *Sorhum bicolor* [27].

The leaf chlorophyll a (Chl-a), chlorophyll b (Chl-b), and carotenoid contents were decreased by 14.41%, 13.81%, and 11.20% respectively. The maximum potential quantum efficiency at the PSII (F_v_/F_m_) ratio was significantly decreased, which was similar to a recent report on wheat [28]. At the same time, in comparison to well-watered mulberry leaf samples, the leaf water content in stressed plants showed a greater reduction of 7.86%, indicating that our plants were indeed experiencing stress. The ratio of electrolyte leakage increased significantly by 2.72-fold after the stress treatment (Figure 1).

### 2.2. C/N Ratio and Ion Content Changes

Sodium accumulated 3.18 and 1.95 folds more in stressed leaves and roots, respectively, of the stressed plants as compared to the plants grown under normal conditions. Potassium content showed no significant changes after the stress treatment. Consequently, the K^+^/Na^+^ ratios were found to decrease in both stressed shoots and roots, especially in the leaves of stressed mulberry plants. The calcium content significantly increased by 23.18% in stressed leaves and decreased by 22.09% in stressed roots. Ca^2+^ is both a nutrient and a signaling molecule in plants. The higher content of Ca^2+^ in the leaves than that in the roots is because in most plants the majority of Ca^2+^ absorbed by roots is transported to the shoots [29]. Ca^2+^ has been shown to participate in multiple processes, such as preserving the integrity of cell membranes [30], stabilizing cell wall structures [31] and regulating ion transport and selectivity [32]. In addition, it has been discovered that plants are able to adjust to high-salt growth conditions by activating a signal transduction system involving Ca^2+^ [33]. Although many studies show that Ca^2+^ content normally increases in response to abiotic stress, a few studies also show that a reduction of Ca^2+^ could also occur depending on the duration of stress [34]. It remains an interesting question to study why the leaf and root have different response to combined stresses in mulberry trees.

The availability of carbon (C) and nitrogen (N) is an important factor in regulating plant metabolism and development [35]. Drought treatment often leads to increased C/N ratio because under this stress normally leaves have increased sugar accumulation and decreased N content [27]. In this present study, the C/N ratio increased with 15.87% and 6.27% in the stressed roots and leaves, respectively (Table 1). The altered C/N ratio, together with the aforementioned physiological parameters, indicated the mulberry plants under our treatment were indeed experiencing drought and salinity stresses.

### 2.3. Identification of Proteins under Salt and Drought Stress

To comprehensively understand the adaptive mechanisms in mulberry, both stressed roots and shoots were sampled and analyzed at the protein level. A total of 309,384 spectra were generated from the TMT experiment using the proteins of salt-drought treated and untreated roots as materials, while 284,360 spectra were generated using leaves as materials, of which 4795 distinct proteins were consistently detected in the present study, including 2944 from the leaves and 4005 from the roots. In terms of the proteins identified in mulberry, 2154 proteins were identified in both the leaves and roots, accounting for 53.78% and 73.17% of the total identified proteins in the roots and shoots, respectively. The false discovery rate was less than 1.72% in the leaf set, and less than 1.87% in the root set (Table 2).

To assess the quantitative precision and reproducibility among the three biological replicates, we applied statistical distribution studies as previous report [36]. The relative expression data (stressed/control) for all the proteins identified for each of the individual treatments were simultaneously fitted to 50 standard data distribution models using EasyFit (MathWave Technologies, http://www.mathwave.com). Data were judged to be the best fit by the Johnson Su distribution, using Kolmogorov/Smirnov, Anderson/Darling and chi-squared tests. The data, including the probability density function, the cumulative distribution function and both the P-P and Q-Q plots, were normally distributed, as indicated in Supplemental Figure S2.

### 2.4. DEPs in Stressed Leaves and Roots

**Figure 2:**
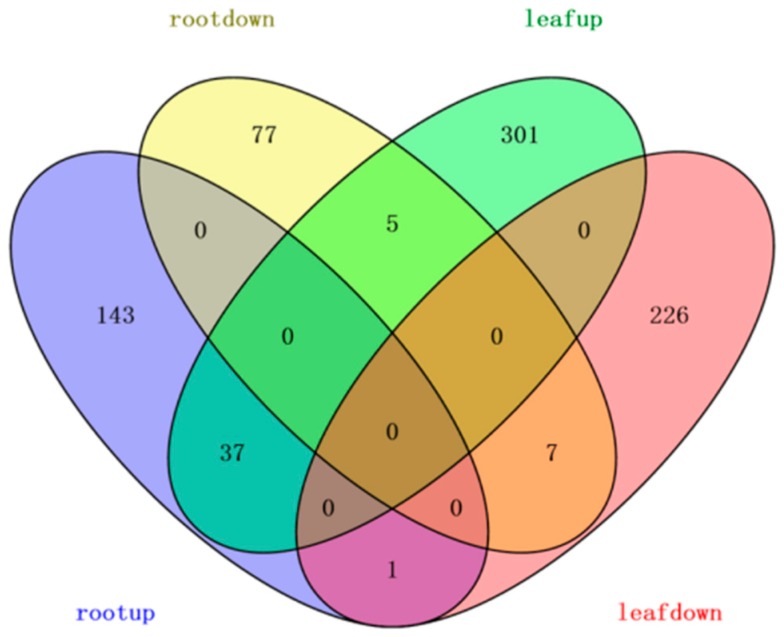
Venn diagram of differentially expressed proteins identified at three different treatment time points.

By calculating the value of the log2 TMT ratio at which 95% of all the proteins had no deviations [36], cut-offs of 1.50- or 0.67-fold were set to determine the upregulation or downregulation of the proteins in the present work. All differentially expressed proteins were positively identified in at least two of the three replicates for each sample. Finally, 343 upregulated proteins and 234 downregulated proteins were detected in leaves, while 181 upregulated proteins and 89 downregulated proteins were detected in roots after the stress treatment (Figure 2). There were more upregulated proteins than downregulated proteins in response to salt-drought stress in the leaves and roots. The overlapping DEPs between the two tissues included 50 proteins represented common changes during the process of salt-drought stress (Table S1).

**Figure 3:**
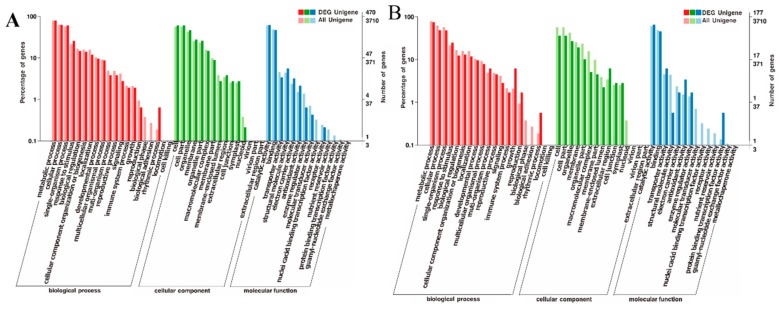
Ontology classification of differentially expressed proteins (DEPs) in the S-D stressed mulberry leaves (**A**) and roots (**B**). The *X*-axis indicates GO terms, while the *Y*-axis indicates the percentage of DEPs.

In the salt-drought stressed mulberry plants, these DEPs assigned to GO (Gene Ontology) classifications fall mostly into three categories: Biological process, cellular component, and molecular function. In stressed leaves, “metabolic process”, “cellular process”, and “single organism process” were the most responsive groups in the biological process category, “cell part”, “cell and organelle” were the most responsive groups in the cellular component category, and “catalytic activity” and “binding” were the most responsive groups in the molecular function category (Figure 3A). In stressed roots, the top three categories of each GO classification in stressed roots mostly overlapped as those in stressed leaves (Figure 3B).

**Figure 4:**
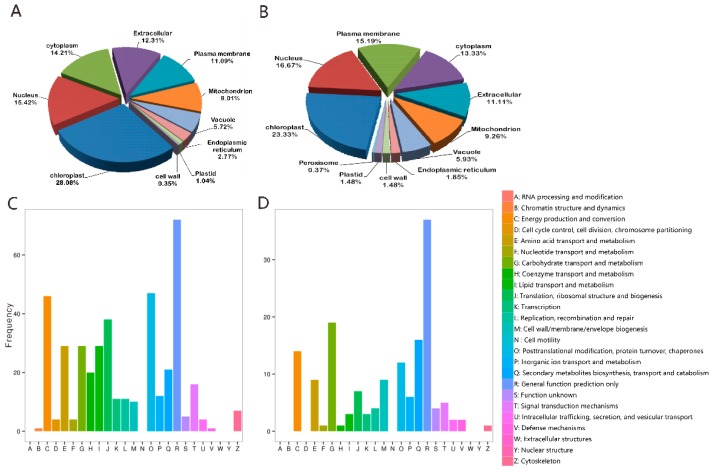
Subcellular localization (upper) and functional categories according to clusters of orthologous groups of DEPs (bottom) in the S-D stressed mulberry leaves (**A**,**C**) and roots (**B**,**D**).

Subcellular localization analysis showed that most of the DEPs are localized in the chloroplast, nucleus, and followed with cytoplasm in leaf samples (Figure 4A) and plasma membrane in root samples (Figure 4B). To understand the function of these DEPs from the perspective of the phylogenetic classification of proteins, 417 leaf DEPs were assigned to 21 COG (clusters of orthologous groups of proteins) categories. The top four most enriched groups were “general function prediction” (72 DEPs), “posttranslational modification” (47 DEPs), “energy production and conversion” (46 DEPs), and “translation, ribosomal structure, and biogenesis” (38 DEPs). Transport and metabolism of “carbohydrate”, “amino acid”, and “lipid” were juxtaposed in the fifth group (Figure 4C). At the same time, the COG analysis assigned 155 root DEPs to 19 categories and the most enriched groups were “general function prediction” (37 DEPs), “carbohydrate metabolite biosynthesis” (19 DEPs), and “secondary metabolite biosynthesis” (16 DEPs) (Figure 4D).

**Figure 5:**
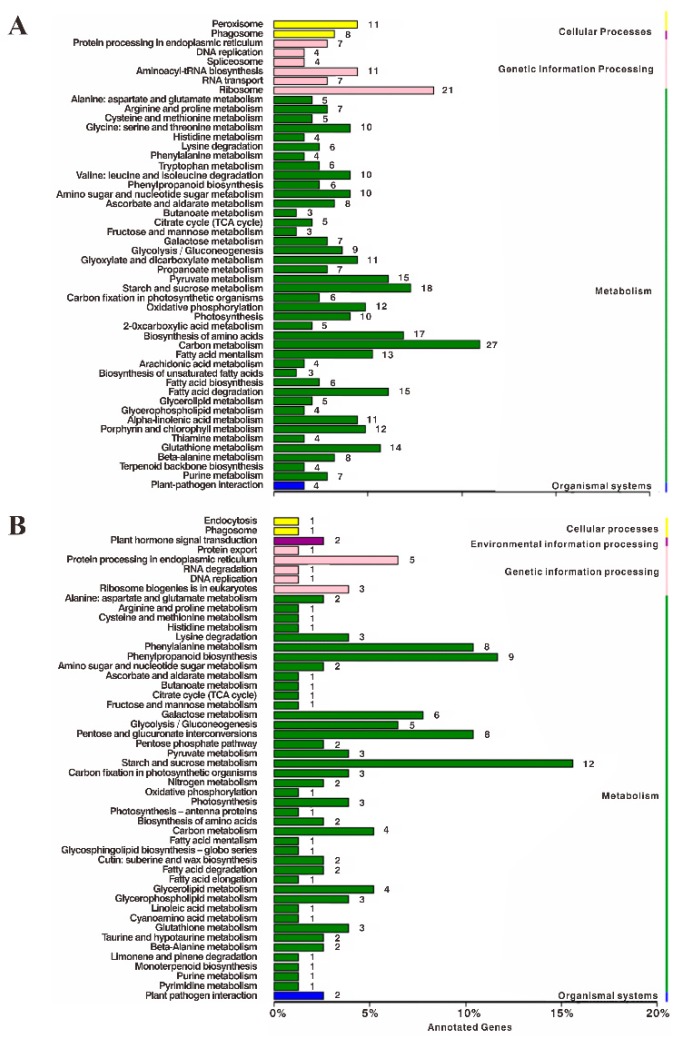
Functional classifications of DEPs in stressed leaves (**A**) and roots (**B**) based on KEGG pathways. The *X*-axis indicates the number of DEGs involved in these pathways. The *Y*-axis indicates the different pathways.

To further understand the function of these proteins from a pathway-specific perspective, we subjected the data to a KEGG (Kyoto encyclopedia of genes and genomes) pathway classification. A lot of 104 different metabolic pathways were revealed, among which the top three enriched categories of pathways of leaf DEPs were “carbon metabolism”, “ribosome”, and “starch and sucrose metabolism”, and the top three enriched categories of pathways of root DEPs were “starch and sucrose metabolism”, “phenylpropanoid biosynthesis” and “pentose and glucuronate interconversions” (Figure 5). The results show that proteins involved in the starch and sucrose metabolic pathway were commonly enriched in leaves and roots under salt and drought conditions, which indicated the importance of these pathways.

### 2.5. Confirmation of Proteomics Alteration by Western Blot

**Figure 6:**
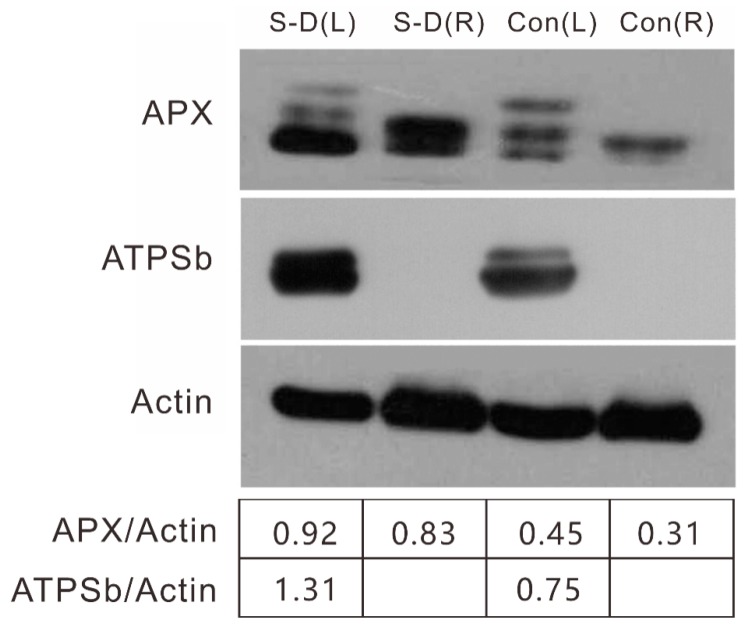
Comparison and validation of two candidate proteins by western blot analysis. Western blot analysis showed changes in APX (ascorbate peroxidase), ATPSb (ATP synthase subunit b), and actin as the internal control. Three replicates were conducted.

To validate the differentially expressed stress-response proteins identified with a proteomics approach in mulberry roots and shoots, immunoblotting was carried out to assess the abundance of the changes of a few representative proteins as determined by TMT proteomics. The concentration of ATP synthase subunit b was reduced in stressed mulberry leaves by western blot, which was in agreement with the proteomics results (Morus013122.p1). APX was measured with upregulation in both mulberry leaves and roots under stress conditions (Figure 6), which was consistent with the alteration of proteins, such as Morus024998.p1, Morus026654.p1 and Morus020267.p1 (Table S2). These immunoblot results were in good agreement with the data from the TMT proteomics analysis and validated the proteomics data.

### 2.6. Mechanisms Response in Mulberry to Combined Stress

According to the physiological analysis and KEGG enrichment results, sugar metabolism and several other typical groups of DEPs were analyzed to help understand the response of mulberry leaves and roots to salt-drought stress.

#### 2.6.1. Photosynthesis and Energy Metabolism

Previous studies have shown that salt or drought stress can affect photosynthesis and enzyme activities associated with CO_2_ assimilation [37]. In the current proteomic study, DEPs related to photosynthesis, photorespiration, and energy were identified in mulberry seedlings exposed to salt and drought stress.

The light reaction is the first stage of photosynthesis to convert light energy into chemical energy, including ATP and NADPH. PSI and PSII are crucial sites for photosynthetic electron transport. In the present study, the level of CP43 (Photosystem II CP43chlorophyll apoprotein, Morus024765.p1) and CP47 (photosystem II47kDa protein, Morus001781.p1) increased under stress. These proteins participate in receiving and transferring photons in PSII. Damage of these proteins could lead to photoinhibition or photodamage under stress [38]. The level of the photosystem Q(B) protein (Morus000029.p1) was also increased. We hypothesized that the increased abundance of these proteins might be responsible for repairing the PSII system under stress, which was similar to the results in a previous report on cucumber [39]. Chlorophyll a-b binding protein (Morus002539.p1), photosystem I P700 chlorophyll an apoprotein A2 (Morus025411.p1), and photosystem I reaction center subunit III (Morus025917.p1) increased in mulberry leaves under salt-drought stress conditions, which indicated that the light reaction proteins were maintained to permit sufficient transfer of excitation energy in stressed mulberry trees.

Proteins involved in photorespiration, including glycerate dehydrogenase (Morus008377.p1), ferredoxin-dependent glutamate synthase (Morus019388.p1), peroxisomal (S)-2-hydroxy-acid oxidase (Morus021638.p1 and Morus021639.p1), and serine-glyoxylate aminotransferase (Morus002102.p1) were upregulated in this experiment. These results suggested a regulation mechanism by increasing energy and reducing the competition involved with the photorespiration process, thereby enabling the coordination of photosynthetic efficiency in mulberry under salt-drought stress conditions.

Under stress conditions, more energy was probably needed in mulberry plants. ATP synthase, which serves as the main enzymes of the ATP biosynthetic pathway and photosynthesis [40], was upregulated in stressed leaves (Morus013122.p1), as shown by our western blotting analysis (Figure 6). The upregulation of ATP synthase has also been observed in wheat [41] and cucumber [39]. An ADP/ATP carrier protein (Morus015275.p1) also increased in stressed mulberry leaves. Four ATPase subunits (Morus019660.p1, Morus002293.p1, Morus008700.p1, and Morus001947.p1) from stressed leaves and ATPase (Morus026914.p1) from stressed roots were identified as increasing in mulberry. As noted in a previous report [42], it is appropriate to assume that H^+^-ATPase plays an essential role in maintaining ion homeostasis in plant cells. The increased abundance of these enzymes might adopt an effective strategy for osmotic adjustment under stress.

#### 2.6.2. Sugar Metabolism

Sucrose is the major form of photoassimilate and is transferred from source leaves to various sink tissues, such as roots. Once unloaded into recipient sink cells, sucrose is cleaved into hexoses by sucrose synthase (SuSy) or invertase (INV) for cellular biosynthesis and metabolism. SuSy catalyzes sucrose and UDP into UDP-glucose and fructose, which play important roles in carbon partitioning and many other important metabolic processes, such as phloem loading, environmental stress response and nitrogen fixation [43]. Here, in the stressed leaves, three sucrose synthases (Morus019552.p1, Morus011123.p1, and Morus011124.p1) were significantly upregulated in the leaves. This increased activity of SuSy enzymes may lead to efficient assimilates supply as described previously in tomato (*cv. Ciettaicale*) [44]. The expression of acid beta-fructofuranosidase (invertase, Morus019026.p1) was downregulated in stressed mulberry leaves. Since this enzyme is targeted in the cell wall and vacuoles, its reduction in mulberry leaves might infer a limited conversion of sucrose into glucose and fructose for development or storage under salt-drought stress conditions. The results indicated that the increase in SuSy and decrease in INV might enable an inner dynamic equilibrium of sucrose cleavage in stressed mulberry leaves and the associated export of carbon and altered shoot to root ratio.

In contrast, in stressed mulberry roots, the acidic beta-fructofuranosidase protein (Morus019026.p1) was downregulated. However, another beta-fructofuranosidase (Morus019157.p1) that functions in the stress defense process in mulberry roots was upregulated in stressed root tissue. Their detailed roles involved in the responses to salt-drought stress remain to be explored. SuSy (Morus019552.p1) was downregulated in stressed mulberry roots, which was a different response from that in the leaves. This result indicated that SuSy isoforms in mulberry have evolved specialized functions in response to salt-drought stress in given organs. Sugar transporter protein13 (STP13) is a member of the monosaccharide transporter (MST) family, which functions as high-affinity hexose-specific H^+^-symporters in transporting apoplast fructose hydrolyzed from sucrose by cell wall INVs. Constitutive overexpression of STP13 in Arabidopsis resulted in seedlings with increased biomass, higher internal sugar levels, more total carbon per plant, and rapid inner nitrate assimilation to accommodate the plant growth rate [45]. In the present study, STP13 (Morus012358.p1) was highly abundant in stressed roots. Considering this information and our aforementioned C/N data (Table 1), although we do not know where this STP transports fructose to, its upregulation is certain to play roles in sugar reallocation in stressed mulberry, e.g., alteration of the total C and N content in mulberry seedlings.

**Figure 7:**
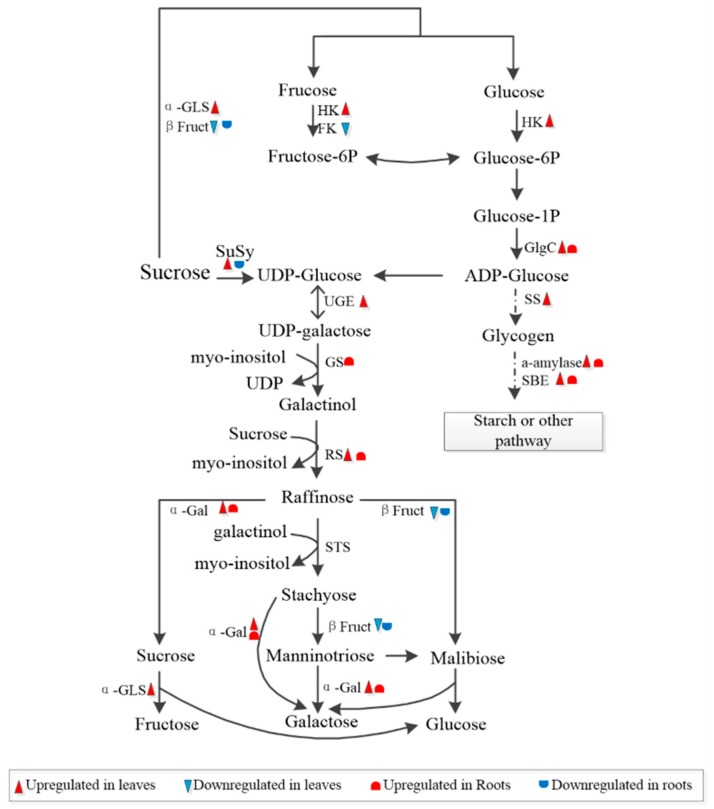
Schematic representation of sugar and glycogen synthesis in mulberry under stress conditions. FK, fructokinase; β Fruct, beta-fructofuranosidase; HK, hexokinase; a-Gal, alpha-galactosidase; GlgC, glucose-1-phosphate adenylyltransferase; a-GLS, alpha-glucosidase; GS, galactinol synthase; RS, raffinose synthase; SBE, 1,4-alpha-glucan branching enzyme; SS, Starch synthase; SuSy, sucrose synthesis; UGE, UDP-glucose 4-epimerase; the upregulated genes are marked in red. (Color figure online).

In addition to sucrose, raffinose family oligosaccharides (RFO) metabolism also showed significant changes in stressed tissues (Figure 7). RFOs are known to function in phloem transport in polymer trapping loading plants, where sucrose is converted into RFOs, a larger sugar, to preclude their transport in the opposite direction [46]. Most plants, such as maize, do not accumulate large quantities of RFOs under optimal conditions. An increase in the production of RFOs has been extensively reported in various plant species in response to seed germination [47,48] and a number of abiotic stresses, e.g., droughts, salinity, and extreme temperatures [49,50,51]. In the present study, raffinose synthase (Morus018010.p1 and Morus017952.p1), which synthesizes raffinose from galactinol and sucrose, and alpha-galactosidase (α-Gal, Morus011230.p1), which hydrolyzes RFOs into sucrose and galactose, were upregulated in stressed mulberry leaves (Figure 7). A UDP-glucose 4-epimerase GEP (Morus002240.p1), which processes UDP-glucose to UDP-galactose, and α-glucosidase (Morus022713.p1), which digests carbohydrates, including starch and stable sugar, were upregulated in stressed mulberry leaves. These results indicated that the accumulation of RFOs was activated via a series of biosynthesis-associated genes in mulberry in response to stress.

In contrast, another raffinose synthase (Morus020545.p1) and α-galactosidases (Morus011230.p1) increased in stressed mulberry roots. In addition, galactinol synthase (GolS, Morus026159.p1) increased in stressed roots. GolS is the first committed enzyme in the RFO synthesis pathway, and it synthesizes galactinol from UDP-galactose and inositol. Transcriptome analyses have long exhibited distinct expression profiles of GolS in different plants under abiotic stress, especially drought and salinity [52,53]. Constitutive overexpression of AtGolS2 conferred drought tolerance and increased grain yield in rice (*Oryza sativa*) genotypes under dry field conditions [54]. One explanation for this scenario might be attributed to premature termination codons (PTCs), which are found interspersed in introns of OsGolS1 and OsGolS2. This PTC is coupled to repress the splicing of regulated unproductive splicing and translation (RUST) under stress and results in adaptation behaviors under stress [54]. The second explanation might be associated with ROS scavenging processes, and phloem mobile signaling compounds under stress [55,56,57]. With significant alterations of enzymes related to the metabolism of RFOs identified in our present study, it is appropriate to assume that the proteins related to RFOs, such as GolS, play roles in adaptation tolerance in mulberry under salt-drought stress conditions. The relationship between these DEPs and sugar allocation in mulberry is unknown. The efficacy and adaptation mechanism of phloem loading under stress remains to be explored in mulberry. Further study of the mechanisms of sugar accumulation and transport in mulberry in response to stress would be interesting.

In addition, starch synthase (Morus011877.p1) and mannitol dehydrogenase (Morus026731.p1) were upregulated in stressed leaves, while glucanases (Morus018091.p1 and Morus09128.p1), which convert cellobiose into glucose, increased in stressed roots. At the same time, glucose-1-phosphate adenylyltransferase (Morus018572.p1), 1,4-alpha-glucan-branching enzyme (Morus024842.p1 and Morus013789.p1), and alpha-glycosyltransferase (Morus011656.p1), which are involved in starch biosynthetic metabolism [58], increased in both mulberry leaves and roots under stress. These results show that alterations in sucrose- and starch-related metabolism are essential for plant development and adaptation mechanisms under abiotic stress responses.

#### 2.6.3. ROS Defense

Under abiotic stress conditions, an excessive quantity of reactive oxygen species (ROS), including superoxide, singlet oxygen, and H_2_O_2_, are produced. Since these ROS molecules are harmful to normal cellular functions, plants have evolved multiple strategies to scavenge them [59]. Glutathione S-transferases (GSTs) represents a major group of detoxification enzymes that play important roles in protecting plants from impairments caused by abiotic stresses. In *Arabidopsis* and grapevines, it has been found that GSTs facilitate the transport of anthocyanins and flavonoids, metabolites of antioxidants under stress [60,61,62]. Seven GSTs (Morus023665.p1, Morus002960.p1, Morus002959.p1, Morus005714.p1, Morus0023833.p1, Morus027945.p1, and Morus018481.p1) and two GSTs (Morus023664.p1 and Morus002961.p1) were upregulated in the leaves and roots, respectively, in the stressed mulberry plants. In addition to the antioxidant protective roles played by GSTs, the activities of a number of antioxidative enzymes, e.g., peroxidases (POD), superoxide dismutase (SOD), catalase (CAT), dehydroascorbate reductase (DHAR), glutathione reductase (GR), monodehydroascorbate reductase (MDHAR), and ascorbate peroxidase (APX), can also be altered to help plants remove ROS species [63]. Two MDHARs (Morus013778.p1 and Morus020520.p1) and two APXs (Morus024998.p1 and Morus026654.p1) and one peroxidase (Morus019790.p1) were increased in the leaves. In addition, eight peroxidase proteins (Morus016288.p1, Morus016289.p1, Morus007537.p1, Morus019224.p1, Morus005917.p1, Morus020176.p1, Morus016735.p1, and Morus018475.p1) were upregulated in stressed mulberry roots. The increased accumulation of these proteins indicated that mulberry plant cells initiated their antioxidant mechanisms to maintain redox homeostasis and resist abiotic stresses.

#### 2.6.4. Ion Homeostasis and Water Transport Metabolism

An excessive amount of Na^+^ interferes with K^+^ uptake by root cells and the competition between Na^+^ and K^+^ ions for binding by diverse enzymes could lead to inhibition of various metabolic processes [64]. It has been documented that K^+^ and Na^+^ transporters play essential roles in salt tolerance by maintaining appropriate K^+^/Na^+^ ratio and ion homeostasis [65]. In this study, potassium transporter 2 (HAK, Morus011233.p1), which is known to function in potassium homeostasis and is crucial for plant survival in saline conditions [66,67], dramatically decreased in stressed mulberry leaves. Similar downregulation pattern of a homologous gene has also been discovered in rice grown under salinity condition [68]. In addition to K^+^, Ca^2+^ also play important roles during the stress response and adaptation against environmental stresses [69]. Free Ca^2+^ level in the cytoplasm is the first reaction in signal transduction in plant response to salt stress. The changed calcium concentration is sensed and decoded by calcium sensor proteins as well as ROS producing enzymes [70]. In the present study, Ca^2+^ binding protein (CBP, Morus027232.p1 and Morus023229.p1) showed declines in concentrations in stressed leaves. In addition, two CBP-EF-hand family proteins (Morus012136.p1 and Morus023835.p1) were upregulated in stressed roots. CBP has been reported to affect drought-responsive element binding transcription factors (DREB2B) and its downstream genes to positively regulate salt tolerance in rice seedlings [71]. Luo et al. reported calcium-binding EF-hand family proteins as cross-talk nodes for salt and drought signaling pathways [72]. These findings suggest that Ca^2+^ associated pathways participate in adaptation responses to salt and drought in mulberry trees.

Aquaporin (AQP) proteins, e.g., PIP (PM intrinsic proteins) and TIP (tonoplast intrinsic proteins) [73], which function in the transport of water and other small solutes through biological membranes, are crucially regulated by developmental and environmental factors [74] In this study, the expression of an aquaporin protein PIP (Morus026380.p1) was increased in stressed mulberry roots. The beneficial effect of having more abundant PIP was demonstrated in studies in which overexpressing PIP1 in *A. thaliana* and *Nicotiana tabacum* plants improved drought resistance [75,76]. TIPs are recognized to facilitate water movement across vacuolar membranes and control the turgor of cells. It is generally believed that the expression of TIPs is positively related to the resistance ability of plants to abiotic stressors. For example, TIP overexpression led to improved drought and salt tolerance in multiple plant species [77,78]. However, an exception has also been found in previous studies. For example, the expression of a *G. soja* TIP gene was repressed in response to abiotic stress. Overexpression of this gene in Arabidopsis led to a compromised resistance ability to drought and salinity [79]. Similar to this discovery, our study shows that the TIP1-3 (Morus010846.P1) was downregulated in stressed mulberry roots. Future more in-depth functional study on this gene using transgenic approach will shed more light on the specific role of mulberry TIP1-3 in relation to abiotic stresses.

#### 2.6.5. Secondary Products

Mulberry trees contain rich flavonoids and other secondary metabolites, such as phenylpropanoids and alkaloids, which have extensive roles in defenses against biotic and abiotic stresses and have long been used in Chinese traditional medicine. Strictosidine synthase (STR, Morus004320.P1), the key enzyme in synthesizing alkaloids [80], was upregulated in stressed mulberry roots. The enzymes 8-hydroxyquercetin-8-*O*-methyltransferase (Morus008922.p1 and Morus000920.p1) and anthocyanidin 3-*O*-glucosyltransferase (Morus022878.p1), which are related to the production of flavanols, such as anthocyanins, were downregulated in stressed roots. Furthermore, two DEPs encoding cytochrome P450s (Morus022050.P1 and Morus004466.P1), which are involved in the regulation of secondary metabolism, were upregulated in stressed roots. These results suggested that the biosynthesis of these secondary metabolites was associated with the salt-stress response in mulberry seedlings.

In addition, proteins involved in the generation of glutamyl-tRNA to chlorophyll, including glutamate-1-semialdehyde 2,1-aminomutase (GSAAT, Morus008822.p1), coproporphyrin oxidase III (Morus012704.p1), tetrapyrrole-binding protein (Morus002377.p1) and uroporphyrinogen decarboxylase (UPD, Morus012310.p1, Morus012311.p1), magnesium-chelatase subunit ChlI (Mg-C, Morus006148.p1, Morus003164.p1) and protochlorophyllide reductase (PPR, Morus025670.p1), showed reduced abundance under salt-drought stress, whereas the expression of pheophorbide an oxygenase protein (PaO, Morus011773.p1), which is a key checkpoint in the overall regulation of chlorophyll degradation, greatly increased in stressed leaves [81]. At the same time, porphobilinogen deaminase (PBGD, Morus007545.p1), which is involved in chlorophyll biosynthesis process, decreased under salt-drought stress conditions. These results indicated that salt stress could inhibit the synthesis of chlorophyll and other tetrapyrroles.

#### 2.6.6. Hormone Metabolism

ABA is a well-known phytohormone that plays important roles in seed germination, seedling growth, stomatal regulation and osmotic stress-responsive gene expression [82]. In this experiment, proteins related to ABA synthesis, including ABA receptor (Morus017591.p1) in stressed mulberry roots, ABA insensitive 5-like (Morus027521.p1) in stressed mulberry leaves, were upregulated. These findings were consistent with a previous report by Rattanakon et al. [83]. Consistent with the biosynthesis of ABA in plants, the enzyme allene oxide synthase (AOS, Morus026982.p1, Morus026991.p1, and Morus011915.p1), catalyzing a-linolenic acid to 12,13(*S*)-epoxy-octadecatrienoic acid (12,13-EOT), which was further processed to 12-oxo-phytodienoic acid (12-OPDA) by the action of allene oxide cyclase (AOC, Morus008669.p1), were upregulated in stressed mulberry leaves. 12-OPDA was a drought responsive regulator of stomatal closure that works more effectively together with ABA [84]. In this experiment, these upregulated-expression proteins were likely playing a role in stomatal closure together with ABA.

Ethylene signaling has also been shown to be involved in drought and salt stress adaptations. Expression of *1-aminocyclopropane-1-carboxylate oxidase (ACO)* genes, the last enzyme in ethylene biosynthesis, is tightly linked to the ethylene levels produced by the plant [85]. The expression level of *ACO* and the production of ethylene were found to be increased in soybean plants grown under drought condition [86]. However, the literature on the involvement of ethylene to salinity stress is contradictory. Although it is generally believed that ethylene plays positive roles in salinity response, a few studies show that higher accumulation of ethylene led to plants with more sensitivity [87,88]. In our study, two mulberry ACO1 (Morus004433.p1, Morus013401.p1) proteins were upregulated in stressed leaves and roots, respectively. This indicated that ethylene plays a positive role in adapting mulberry to cope with the drought and salinity adverse environment. Future transgenic studies with mulberry genes related to ABA and ethylene biosynthesis will delineate a firmer relationship between hormonal production and stress tolerance.

## 3. Materials and Methods

### 3.1. Plant Material

Mulberry seedlings (You 1#) seeds were sown in pots filled with garden soil and grown in an illuminated chamber (Percival, IA, USA) with 400 μmoL m^−2^ s^−1^ photosynthetically active radiation, a 14 h/10 h (day/night) cycle, and a day/night temperature of 25/22 °C at a relative humidity of 75%. Two months later, 30 mulberry seedlings were randomly divided into two groups, i.e., the wild type control (WT) and salt-drought stress treatment (S-D). Each group contained three independent biological replicates (labeled WT1~3 and S-D1~3) and each replicate contained five independent mulberry seedlings. The S-D group was irrigated with 200 mM NaCl for two days to impose salinity stress [89], drought stress of the same group of salinity treated plants was initiated by withdrawing water in the following two weeks. The WT group was watered normally every day. At the end of the treatment, the leaves and roots from each mulberry seedling were separately collected and snap frozen in liquid N_2_, and stored at −80 °C, respectively.

### 3.2. Physiological Analysis

Chlorophyll fluorescence was measured using an imaging system PAM 101 (Walz, Effeltrich, Germany). The maximum quantum efficiency of PSII (F_v_/F_m_) was calculated according to the manufacturer’s instruction. Chlorophyll was extracted using a 95% alcohol solution, and the chlorophyll a and b contents were measured using a spectrophotometer, as described by Habib [90]. Ion leakage expressed as a percentage was determined using a conductivity meter (Leici-DDS-307A, Shanghai, China), as described by Ai et al. [91]. The elemental content, including Na, K and Ca, in both the shoots and roots was determined by dry ash extraction methods as described by the Cornell nutrient analysis laboratory (http://cnal.cals.cornell.edu/). Total C and total N contents were analyzed by element analyzer (Weipu Ltd., Shanghai, China).

### 3.3. Protein Extraction, Digestion, and TMT Labeling

Proteins were first extracted from leaves and roots using the phenol extraction method and then precipitated by the ammonium acetate-methanol approach [92]. Concentrations in all extracts were determined using a Bio-Rad protein assay kit (Bio-Rad, Hercules, CA, USA) with bovine serum albumin as the protein standard. Further quantification was conducted on a precast NOVEX 12% Tris/Glycine mini-gel (Invitrogen, Carlsbad, CA, USA) along with a series of amounts of *E. coli* lysates (2.5µg/lane, 5 µg/lane, 10 µg/lane, 20 µg/lane) (Figure S3). The SDS gel was visualized with colloidal Coomassie blue stain (Invitrogen, CA, USA), imaged by Typhoon 9400 scanner, and analyzed by Image Quant Software version 8.1 (GE Healthcare).

Proteins were identified and quantified at the Cornell MS Facility, using a TMT-based comparative proteomics analysis method. An overview of the experimental design and workflow is shown in Figure S4. The digested peptides (100 µg each sample) were labeled with TMT 6-plex reagents. The leaves from the WT1~3 and S-D1~3 treatments were labeled as 126-tag, 127-tag, 128-tag, 129-tag, 130-tag and 131-tag in one set, while the roots from the WT1~3 and S-D1~3 treatments were labeled in a second set. The efficiency of TMT 6-plex labeling was assessed by MALDI-TOF/TOF 4700 (AB Sciex, Framingham, MA, USA). After labeling, the six samples were pooled and run through PolyLC strong cation-exchange cartridge (PolyLC Inc. Columbia, MD, USA) and desalted by Sep-Pak SPE cartridges (Waters, Milford, MA, USA) for subsequent high-pH reverse phase (hpRP) fractionation separation.

### 3.4. High PH Reverse Phase (hpRP) Fractionation and NanoLC-MS/MS Analysis

The hpRP chromatography was carried out using a Dionex UltiMate 3000 HPLC system with the built-in micro fraction collection option in its autosampler and UV detection (Sunnyvale, CA). The TMT 6-plex tagged tryptic peptides were reconstituted in buffer A (20 mM ammonium formate pH 9.5), and loaded onto an XTerra MS C_18_ column (3.5 µm, 2.1 × 150 mm, Waters, Milford, MA, USA) with 20 mM ammonium formate (NH_4_FA), pH 9.5 as buffer A and 80% ACN/20% 20 mM NH_4_FA as buffer B. Forty-eight fractions were collected at one-minute intervals and pooled into a total of 12 fractions based on the UV absorbance at 214 nm and with multiple fraction concatenation strategy. All of the fractions were dried and reconstituted in 2% ACN/0.5% FA for nano-liquid chromatography (LC)-MS/MS analysis in an LTQ-Orbitrap Velos (Thermo-Fisher Scientific, San Jose, CA, USA) mass spectrometer equipped with a “CorConneX” nano-ion source (CorSolutions LLC, NY, USA) as reported previously [93].

### 3.5. Data Processing, Protein Identification, and Auantification

Raw data files acquired from Orbitrap were converted into MGF files using proteome Discover 1.4 (Thermo-Fisher Scientific, Bremen, Germany). Subsequent database searches were performed with the 2.3.02 version of the Mascot software (Matrix Science, Boston, MA, USA). Protein identification was performed against the *Morus* genome protein database (http://morus.swu.edu.cn/morusdb/) [21]. The default search settings included one mis-cleavage for full trypsin with a fixed carbamidomethyl modification of cysteine, fixed 6-plex TMT modifications on lysine and N-terminal amines, variable modifications of methionine oxidation, and a deamidation on asparagine/glutamine residues. The peptide mass tolerance and fragment mass tolerance values were 20 ppm and 100 mDa, respectively. To estimate the false discovery rate (FDR) and measure the identification certainty in each replicate set, we employed the target-decoy strategy of Elias and Gygi in Mascot [94]. Specifically, an automatic decoy database search was performed in Mascot by choosing the decoy checkbox, in which a random sequence from the database was generated and tested for the raw spectra and the true database. To reduce the probability of false peptide identification, we considered only those significant scores at a 99% confidence interval for the peptides defined by a Mascot probability analysis (www.matrixscience.com/help/scoring_help.html#PBM). Each case required at least two unique peptide identifications, as indicated in Mascot. The quantitative protein ratios were weighted and normalized by the median ratio with outlier removal set as automatic in Mascot for each set of experiments. The manufacturer’s recommended isotope correction factors were applied.

### 3.6. Functional Category and Clustering Analysis

Based on Cutoff statistics from each replicate [36], the fold change of differentially expressed proteins (DEPs) was set as 1.5. The identified proteins are annotated to multiple databases, including NR (NCBI non-redundant protein sequence), COG, KEGG, and GO. GO enrichment analysis of the DEPs was implemented by the top GO R package-based Kolmogorov–Smirnov test [95]. Subcellular location of DEPs was predicted using SherLoc2 (http://abi.inf.uni-tuebingen.de/Services/SherLoc2).

### 3.7. Antibodies and Western Blot Analysis

The protein extracts from the mulberry leaves and roots (100 μg each sample) were separated on 12.5% SDS-PAGE gels and electroblotted onto a PVDF membrane using a Mini Trans-Blot Cell (BioRad, Foster City, CA, USA). Membranes were blocked and incubated with antisera of one of the following proteins: Monoclonal anti-actin (plant) antibody produced in mouse (purchased from Sigma-Aldrich, St. Louis, MO, USA, product Cat: A0480), ATP synthase subunit b or ascorbate peroxidase 3. Goat anti-rabbit IgG conjugated with alkaline phosphatase was used as the secondary antibody, and the bands were visualized with a premixed NBT substrate solution (Sigma-Aldrich, St. Louis, MO, USA).

### 3.8. Statistical Analysis

The significance of multiple comparisons among the groups was tested with one-way ANOVA, followed by Duncan’s multiple range tests via SPSS statistical software (version 22.0, SPSS, Inc. Chicago, IL, USA). Data were presented as the mean ± standard deviation of three replicate samples. Statistical significance was considered at *p* ≤ 0.05.

## 4. Conclusions

Soil salinity and drought are the two most common abiotic stresses that limit plant growth and productivity throughout the world. Mechanistic understanding of either of these stresses individually has been studied in multiple plants. However, very little is known about the global response of leaves (source) and roots (sink) under the combined stress conditions. Here, we provide the first profile of physiologic proteomics in terms of adapting to salt-drought stress at the whole-plant level in mulberry. Our results show that the stressed mulberry had significant changes in its root/shoot ratio, electrolyte leakage, and water content. Those differential expressed proteins were greatly related to carbon metabolism, photosynthesis, ROS defense, secondary metabolism, and hormone metabolism, which synergistically play roles in the response of salt-drought stress in mulberry trees. This study provides information on the global adaptation response in mulberry trees under a combination of salt and drought stress and provides a basis for further genetic engineering and crop improvements.

## Figures and Tables

**Table 1:** Changes in R/S, C/N and ion content in stressed mulberry tissues.

Samples		R/S Ratio ^1^	Total C (%)	Total N (%)	C/N Ratio	Na^+^ (g/Kg)	K^+^ (g/Kg)	Ca^2+^ (g/Kg)	K^+^/Na+ Ratio
**Con**	Leaf	0.12 ± 0.03	40.66 ± 0.20	4.90 ± 0.09	8.29 ± 0.18	0.45 ± 0.05	19.94 ± 0.46	20.75 ±0.85	44.59 ± 4.17
	Root		40.11 ± 0.09	4.33 ± 0.06	9.26 ± 0.11	0.81 ± 0.08	17.43 ± 1.85	2.49 ± 0.17	21.71 ± 4.25
**S-D**	Leaf	0.26 ± 0.04 **	38.84 ± 0.21 **	4.41 ± 0.05 *	8.81 ± 0.14	1.43 ± 0.12 **	21.68 ± 0.11	25.56 ± 1.14 **	15.20 ± 1.22 **
	Root		41.84 ± 0.21 **	3.91 ± 0.21	10.73 ± 0.52	1.58 ± 0.01 **	16.23 ± 1.14	1.94 ± 0.10 **	10.28 ± 0.70 **

^1^ R/S ratio stands for dry weigh biomass comparison between the roots and shoots. Data from stressed samples were compared with that from control group. Significant differences with *p* < 0.05 were marked with *, while differences with *p* < 0.01 were marked with **.

**Table 2:** Primary results for identified proteins from mulberry leaves and roots.

Protein Category	Leaf	Root
Total spectra	284,360	309,348
Peptides identified	49,758	64,317
Protein identified	3615	4768
Number of unique proteins	942	2095
Combined distinct protein IDs (total/overlap) from two tissues	5710/2673	
Total proteins IDs with TMT ratio	2944 (81.44%)	4005 (84.00%)
Unique proteins with TMT ratio	790	1851
Combined distinct protein IDs with TMT ratio (total/overlap) from two tissues	4795/2154	

## Supplementary Materials

Supplementary materials can be found at https://www.mdpi.com/1422-0067/20/10/2486/s1. **Figure S1.** Soil moisture and salinity at the end of the test treatment. **Figure S2.** Comparison of the relative expression data (treated/control on average) for all proteins quantified for each of the individual treatments in three biological replicates. (**A**)&(**E**). Probability density function of each replicate data (**A**, leaves; **E**, Roots) was simultaneously fit to 50 standard data distribution models using the EasyFit software (huup://www.mathwave.com). The data were judged to be best fit by Johnnson Su distribution. (**B**)&(**F**). Cumulative distribution function (CDF) for all identified proteins in three biological replicates in both leaves (**B**) and roots (**F**). Consistent distribution for each replicate data was demonstrated. (**C**)&(**G**). Probability-probability (P-P) plot of each replicate data (**C**, Leaves; **G**, Roots) were described. This P-P plot was graph of the empirical CDF values plotted against the theoretical CDF values, and used to determine how well a specific distribution fits to the observed data. The approximately linear plots for each data confirmed the correct theoretical distribution model and data consistency. (**D**)&(**H**). Quantile-quantile (Q-Q) plot is described and verified as a graph (**D**, Leaves; **H**, Roots) of the input data (observed) values plotted against the theoretical (fitted) distribution. **Figure S3.** Images from SDS gel electrophoresis of mulberry proteins used in the proteomics study. (**A**) Samples from stressed leaves and roots. (**B**) Samples from the control group. Note: 1 μL of each sample was loaded onto the gel. **Figure S4.** Schematic diagram for the proteomics workflow conducted in this study. **Table S1.** Classification of the DEPs commonly identified from stressed leaves and roots in mulberry. **Table S2.** Classification of DEPs in response to salt-drought stress in mulberry.
