# Supplementary material for: Physiological and Proteomic Responses of Mulberry Trees (Morus alba. L.) to Combined Salt and Drought Stress
Source: Int J Mol Sci. 2019 May 20;20(10):2486. doi: 10.3390/ijms20102486 (PMC6566768; doi:10.3390/ijms20102486)
Supplement: Supplementary file 1 [file ijms-20-02486-s001.zip › ijms-477862-supplementary/Supplementary figures.docx]

**Supplementary figures**





**Figure S1.** Soil moisture and salinity at the end of the test treatment.

**
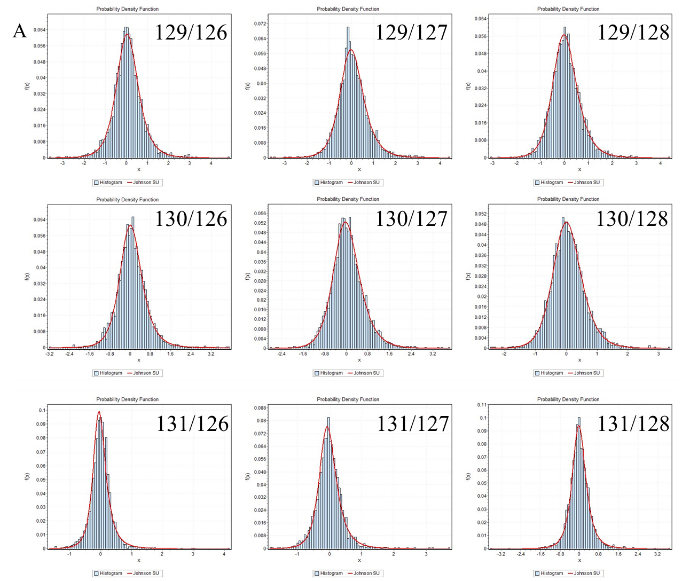

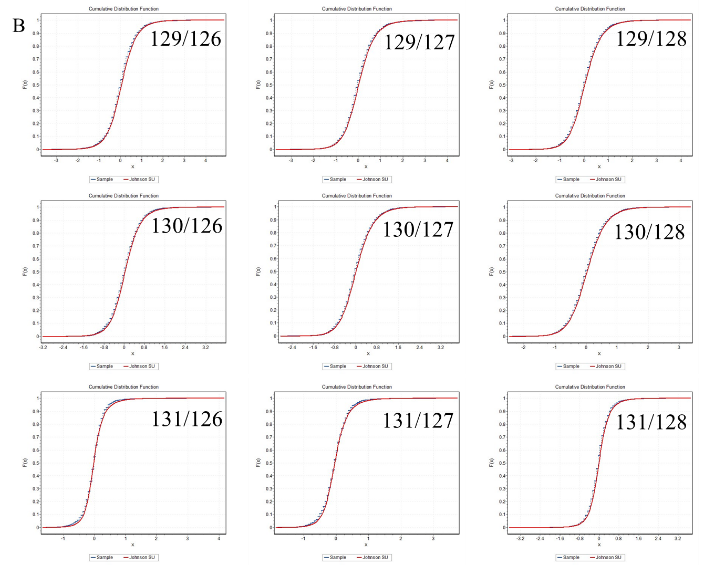
**

**
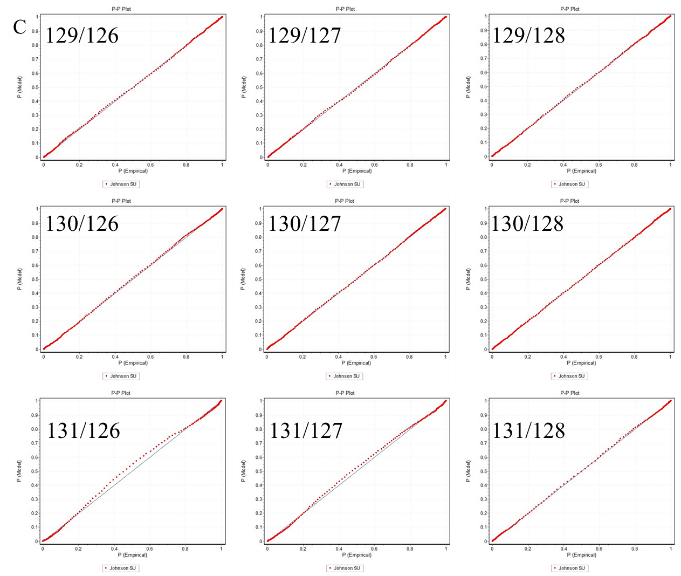

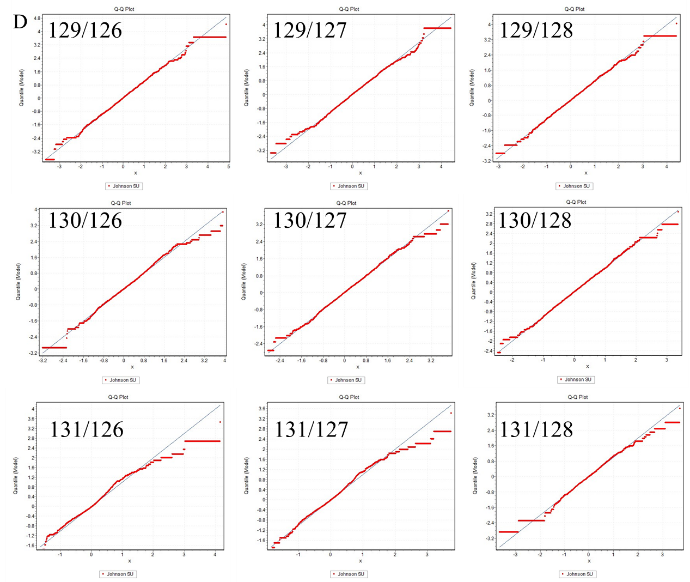
**


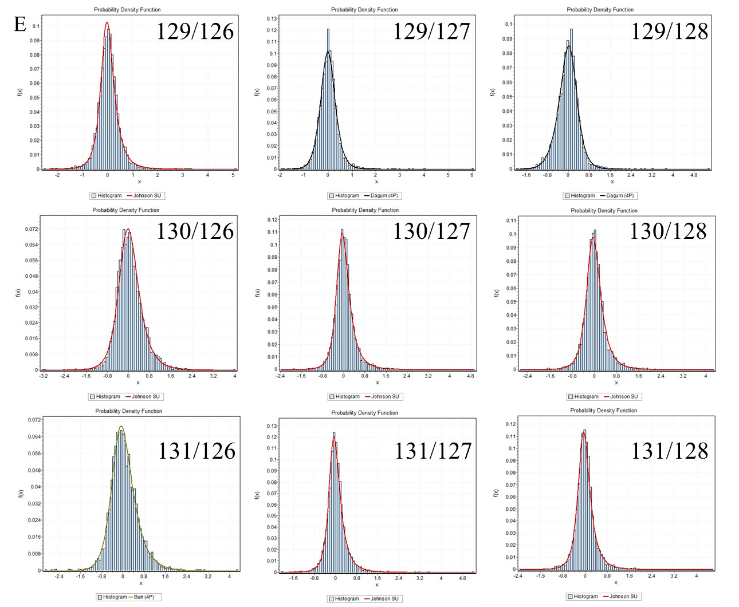

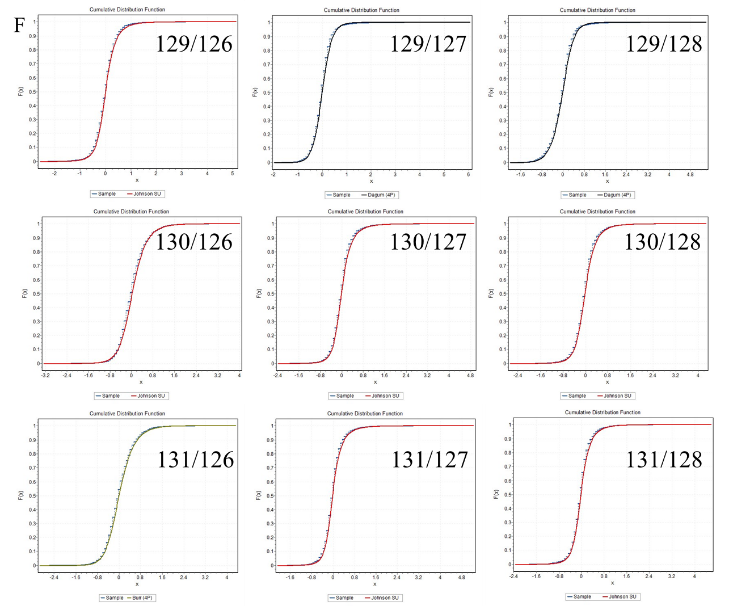

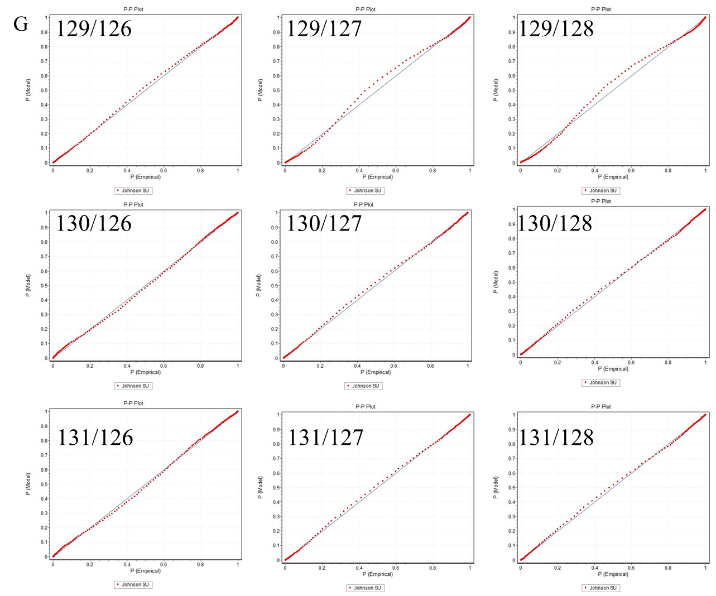

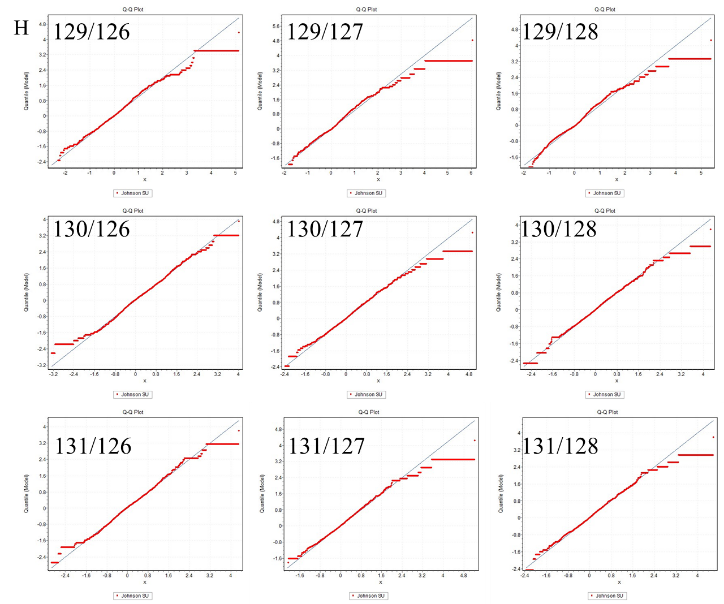


**Figure S2.** Comparison of the relative expression data (treated/control on average) for all proteins quantified for each of the individual treatments in three biological replicates. (**A**)&(**E**). Probability density function of each replicate data (**A**, leaves; **E**, Roots) was simultaneously fit to 50 standard data distribution models using the EasyFit software (huup://www.mathwave.com). The data were judged to be best fit by Johnson Su distribution. (**B**)&(**F**). Cumulative distribution function (CDF) for all identified proteins in three biological replicates in both leaves (**B**) and roots (**F**). Consistent distribution for each replicate data was demonstrated. (**C**)&(**G**). Probability-probability (P-P) plot of each replicate data (**C**, Leaves; **G**, Roots) were described. This P-P plot was graph of the empirical CDF values plotted against the theoretical CDF values, and used to determine how well a specific distribution fits to the observed data. The approximately linear plots for each data confirmed the correct theoretical distribution model and data consistency. (**D**)&(**H**). Quantile-quantile (Q-Q) plot is described and verified as a graph (**D**, Leaves; **H**, Roots) of the input data (observed) values plotted against the theoretical (fitted) distribution.


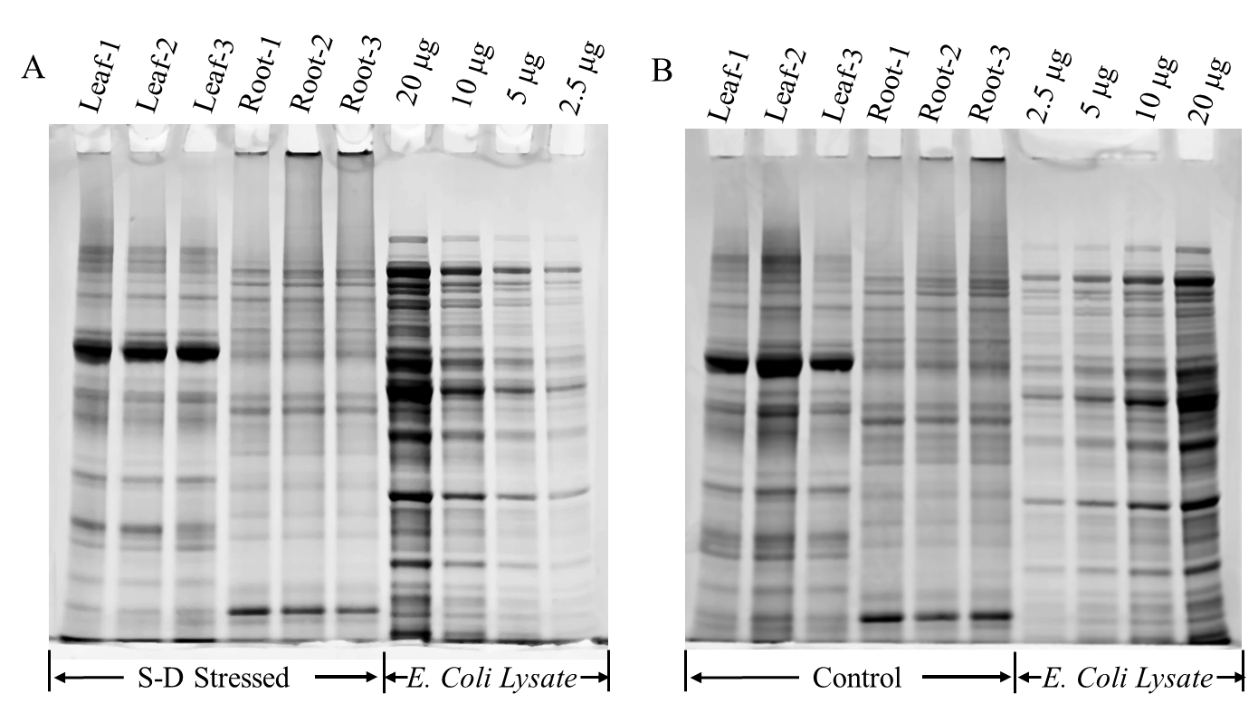


**Figure S3.** Images from SDS gel electrophoresis of mulberry proteins used in the proteomics study**.** (**A**) Samples from stressed leaves and roots. (**B**) Samples from the control group. Note: 1 μl of each sample was loaded onto the gel.


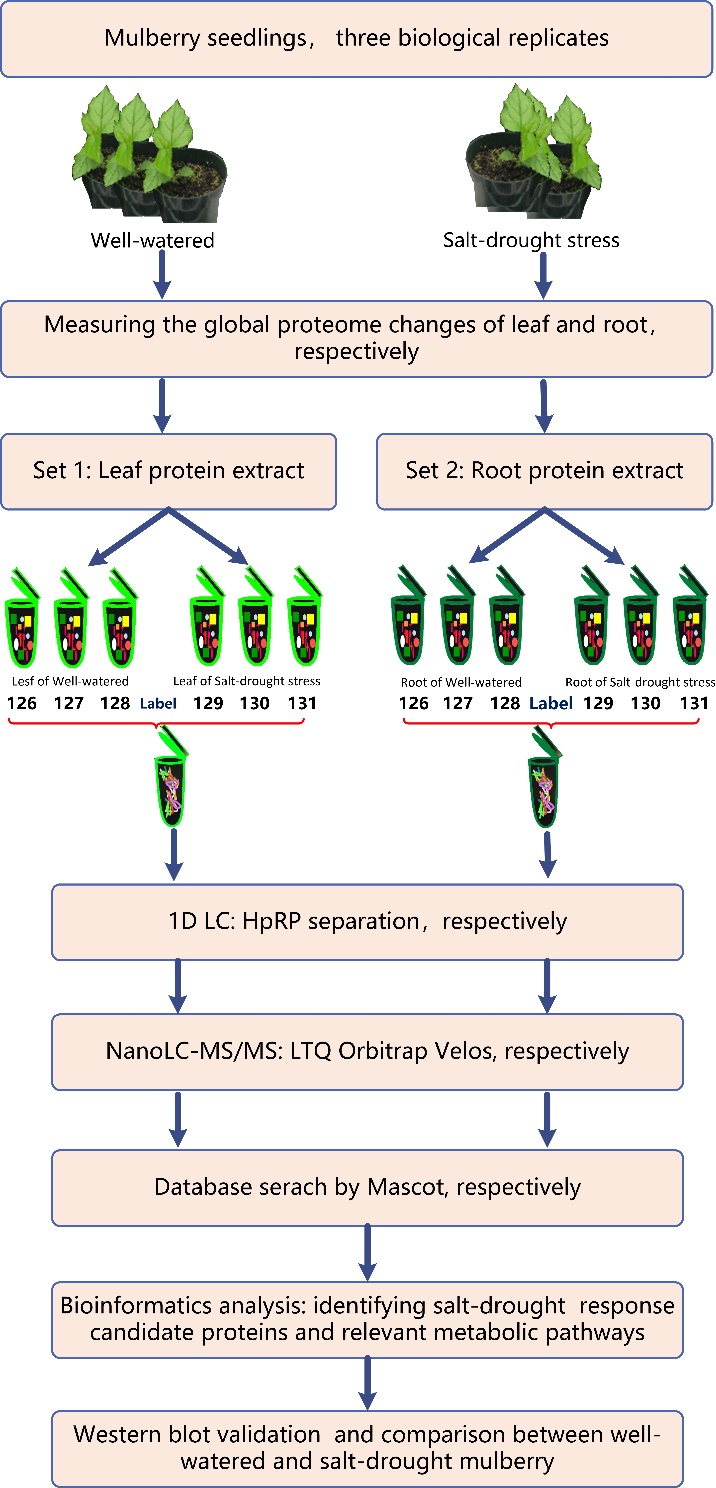


**Figure S4.** Schematic diagram for the proteomics workflow conducted in this study.
